# Appreciated, with Much Thanks

**Published:** 1888-12

**Authors:** 


Appreciated, with much Thanks.—The Buffalo Medical and Surgical Journal is out in a charming autumnal dress. The profession of the Empire State can well feel proud of this metropolitan periodical.—*Southern California Practitioner.*

